# *Fc Receptor-Like* Gene Expression in Renal Transplantation Patients

**DOI:** 10.31661/gmj.v9i0.1730

**Published:** 2020-09-12

**Authors:** Narges Jamshidian Tehrani, Zahra Amirghofran, Ali Reza Shamsaeefar, Aida Karachi, Mohammad Hossein Karimi

**Affiliations:** ^1^Organ Transplant Research Center, Shiraz University of Medical Science, Shiraz, Iran; ^2^Shiraz University of Medical Science, Shiraz, Iran

**Keywords:** Fc Receptor-Like Molecules, Kidney Transplantation, Peripheral Blood Mononuclear Cells

## Abstract

**Background:**

It has been well-documented that the Fc receptor-like (FCRL) molecule contributes to the pathogenesis of certain autoimmune disorders. FCRL molecules belong to the immunoglobulin superfamily produced by B cells. Also, these molecules induce activating or inhibitory signals of B cells. According to this information and also considering the critical role of immune reactions in organ transplantation, the following experiment was performed to analyze the gene expression level of *FCRLs* in peripheral blood mononuclear cells of kidney transplant recipients.

**Materials and Methods:**

Blood samples were obtained from 32 renal transplant patients on days 1, 3, and 7 post-transplantations. Patients were divided into two groups according to the presence or absence of rejection. Also, 24 age-matched healthy subjects were enrolled as control group. After total RNA extraction from peripheral blood mononuclear cells (PBMC) and cDNA synthesis, the gene expression levels of *FCRL1*, *FCRL2*, and *FCRL4* in each group were measured by real-time polymerase chain reaction.

**Results:**

Our results showed that *FCRL1* expression levels in kidney transplant patients were significantly less than healthy controls. The overall *FCRL2* expression level was not significantly different between them. However, at days 1 and 7, following transplantation in the non-rejected group *FCRL2* level was significantly higher than the control group. Comparing the *FCRL4* gene expression levels of both groups with healthy controls showed a significant decrease in the third and seventh days post-transplantation.

**Conclusion:**

It can be concluded that mononuclear cells, mainly B cells, have an essential role to play in kidney transplantation.

## Introduction

Nowadays, renal transplantation has become a well-accepted therapy for patients with end-stage renal disease [1–4]. After solid organ transplantation, the production of donor-specific antibodies (DSAs) increases and causes rejection [5]. B lymphocytes have a major contribution to the balance of transplant rejection [6]. B cells are considered to increase the humoral immune response because of their potential for antibodies production [7]. Although antibody-mediated rejection (ABMR) is the major reason for allograft loss [8,9], B cells can promote allograft rejection as an antigen-presenting cell (APC) or through the production of DSAs [10]. However, the function of B cells is affected by numerous molecules with different properties. Some of these molecules have been recognized to be capable of increased responsiveness of the immune system. Fc receptor-like ( *FCRL*) molecules are an important family with alternative names, including IFGP, SPAP, FCRH, and IRTA [11]. *FCRL* molecules are related to the Fc receptor (FCR) gene family by structural, genomic organizational, and chromosomal position [12]. In human beings, the *FCRL* family includes eight genes located on chromosome 1q 21-23. *FCRL*s 1-6 transmembrane glycoproteins consists of Ig-like domains immune receptor tyrosine-based inhibitory motifs (ITIM) and/or tyrosine-based activation motifs (ITAM) [11,13,14]. However, phylogenetic of FCR and *FCRL 1–5 *molecules to be of five, unlike subtypes [12,15]. *FCRLs 1-5 *are expressed mainly by the B cell lineage. Today, the expression of *FCRL* molecules has been studied in some malignancies and infections [16,17]. Association of *FCRL3*, with autoimmune diseases such as rheumatoid arthritis (RA), multiple sclerosis, and Graves’ disease (GD), has been reported recently [18]. The reports are limited to the expression profile of the *FCRL* family in renal transplantation. We attempted to investigate the expression patterns of *FCRL1*, *FCRL2*, and *FCRL4 *molecules at the mRNA level in peripheral blood mononuclear cells (PBMC) derived from renal transplanted patients.

## Materials and Methods

###  Patients and Control Subjects

Three EDTA-treated blood samples were taken from the patients at first, third, and seventh days post-transplantation. PBMCs were isolated by Ficol (lymphodex, Germany) density gradient centrifugation. PBMCs were separated from EDTA-treated blood samples and subsequently stored at -80°C until all the samples were collected. Blood samples were divided into two groups, including rejection (6 patients) and non-rejection (26 patients). Furthermore, the present study used 24 healthy subjects as a control group. The control group’s age and sex were matched with the healthy controls to compare the expression levels of *FCRL*. The control group displayed no autoimmune diseases. The Ethical Committee of Shiraz University of Medical Sciences approved the present study (ethical code:12593).

###  RNA Extraction and cDNA Synthesis

 TRIzol reagent (Invitrogen, USA) was used to extract total RNA from the samples according to the manufacturer’s instruction. A NanoDrop spectrophotometer (Thermo Scientific, USA) was applied to evaluate the concentration of RNA (adjusted to 250 ng/μl). Subsequently, cDNA was synthesized using a cDNA synthesis kit (Takara, Japan), according to the manufacturer’s instruction. The cDNA obtained in this study was stored at -20°C until used for real-time polymerase chain reaction (PCR) experiments.

###  Quantitative Real-Time PCR

Real-time PCR was used to determine *FCRL1*, *FCRL2*, and *FCRL4* genes in the patients and controls. The human glyceraldehyde-3-phosphate dehydrogenase gene ( *GAPDH*) was applied as a housekeeping gene or internal control. Table-1 indicates specific primers used for real-time PCR. The PCR was carried out using SYBR®Premix (Takara, Japan) with the Real-time PCR System (ABI step one plus, Applied Biosystems, USA). PCR was conducted in a final volume of 20 μl containing a 2-μl cDNA template, forward and reversed primers, SYBR Premix, ROX Reference Dye II, and dH2O. Table-1 showed the PCR cycle programs used; also, to validate specific amplification, each reaction’s melting curves were monitored. The 2−ΔΔCT formula determined the relative fold changes in *FCRL* gene expression of the patients and controls.

###  Statistical Analysis

The analysis of the collected data was conducted using the nonparametric Mann–Whitney U test. The mean ± standard error of the mean (SEM) was used to measure differences between the two groups. The Spearman correlation test was applied to evaluate the correlation between *FCRL* gene expression levels and clinical trials. Statistical analyses were carried out using SPSS version 19 (IBM, USA). P-values less than 0.05 were considered to be significant.

## Results

**Figure 1:**
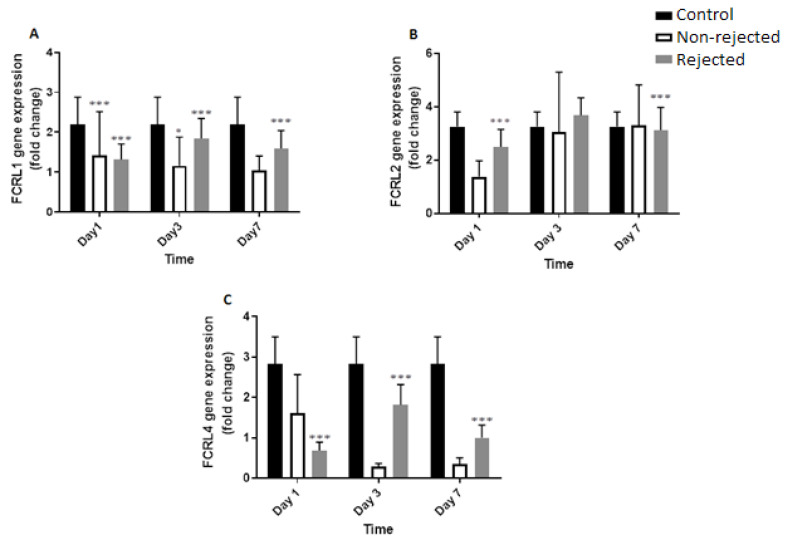
**Figure 1. **Gene expression levels (fold change) of FCRL1(A), FCRL2 (B), and FCRL4 (C) at 1st, 3rd, and 7th days.
 *P<0.05 vs. control, ***P=0.0001 vs. control

The non-rejection group included 26 patients, containing seven females (27%) and 19 males (73%), ranging from 26 to 74 years old (mean of 51.62 ± 10.6 years). The rejected group included six patients, containing one female (15%) and five males (85%), ranging from 27 to 69 years old (mean of 50.95 ± 10.61years). Blood group O+ exhibited the most frequent ABO blood group in both patient groups. Table-2 shows patient demographics and laboratory tests conducted for each group. As shown in Table-3 and Figures-1, *FCRL1* gene expression in days 1, 3, and 7 of both non-rejection and rejected patients differ significantly from the control group (P=0.0001). However, no significant differences were found in *FCRL2 *gene expression compared with the control group except that of the non-rejection group showed a significant difference in days 1 and 7 (P=0.0001). However, significant differences were detected in the *FCRL4 *gene expression in the non-rejection group in days 1, 3, and 7 (P=0.0001).

## Discussion

*FCRL* molecules are indicated as a receptor family wholly expressed by lymphocytes, mainly B cells that play critical regulatory roles in responses and development of B cells [19]. Signaling pathways of B cell receptors might be making the immunomodulation of their responses, autoimmune or immunodeficiency diseases [20,21]. In the present study, the expression levels *FCRL1* gene were assessed using real-time PCR in PBMCs derived from kidney transplant rejected and non-rejection patients and compared with the control group. Additionally, we focused mainly on *FCRL1*, *FCRL2*, and *FCRL4* because of the presence of two and three ITAMs in the cytoplasmic region of *FCRL1* and * FCRL4* them enhancer and inhibitory receptors, respectively [19]. *FCRL2* has two ITIMs and a cytoplasmic domain that it may have two function receptors. On the other hand, a mutational investigation recommended that the B cell response parameter has a negative immunomodulatory function of *FCRL2* [20]. The expression levels of * FCRL*s genes have been studied in autoimmune diseases, such as Hashimoto’s thyroiditis (HT), GD, and RA [22-24]. The present study investigated *FCRL1*, *FCRL2*, and *FCRL4 *genes expression in patients with kidney transplanted. As our results showed, there were significant differences in the *FCRL1 *gene expression in both rejecting and non-rejecting groups. However, *FCRL2* gene expression showed no significant alteration except for the non-rejecting group showing a significant difference; furthermore, a significant difference was found in the expression level of the *FCRL4* gene in the non-rejecting group on days 1, 3, and 7 with compare to the control group. In some studies, it has been demonstrated that the *FCRL1* gene expression levels expression in patients with multiple sclerosis, lupus anticoagulants, arteritis, and von Willebrand disease are higher than that of healthy subjects [21]. This finding suggested that *FCRL1* might play a critical role in kidney transplant pathogenesis or allograft rejection. In a previous study, two other autoimmune disorders, HT and GD, showed a significant decrease in the *FCRL1* gene expression level but a considerable increase in *FCRL2* and *FCRL4 *genes expression with compare to the corresponding healthy controls [22]. * FCRL4 *was expressed in significantly lower levels in patients with kidney transplantation than those of the control. Yeo *et al*. [18] reported the involvement of * FCRL4* in RA. Besides, they introduced a new subset of B cells capable of expressing *FCRL4* with a different pro-inflammatory and bone destructive cytokine pattern in the rheumatoid synovium. Accumulating evidence indicated that this subset of B cells is a pathogenic B cell subset in kidney reject transplanted. Although *FCRL2 *and *FCRL4* are most likely expressed by memory B cells, *FCRL4* is expressed mainly on a unique subset of memory B cells identified by the IgD-CD27-phenotype [23,24]. *FCRL2 *expression has been suggested to be a negative regulator for B cell [20]; therefore, its higher expression could be a compensatory mechanism to decrease B cell function. However, further studies are required to determine the *FCRL* signaling pathways and find its relation to rejection or non-rejection of kidney transplantation.

## Conclusion

Previous findings and our results demonstrated the potential roles of *FCRL* molecules in graft survival. *FCRL1*, *FCRL2*, and *FCRL4 *are suggested to be critical elements in the graft’s immunological processes. It can be concluded that mononuclear cells, mainly B cells, play important and effective roles in kidney transplantation through the *FCRL* pathway.

## Acknowledgment

 This study was supported by the Organ Transplant Research Center (grant number:96/232).

## Conflict of Interest

 The authors declare no potential conflicts of interest.

**Table 1:** List of Specific Primers Used in this Study.

**Genes**	**primers**	**Primer sequences (5’-3’)**	**Amplicon size**	**Thermo cycling conditions**
*FCRL1*	F R	GGTCATACTGGTGCGAGGCAC CAGATGAGGACCAGCCT	157	95°C/30 s, 95°C/15.s, 40 cycles of 58°C/20s and 72°C/30 s
*FCRL2*	F R	GTATGTCAATGTGGGCTCTG TCTGATTCCTCCAAGTGTTATG	162	95°C/30 s, 95°C/15.s, 40 cycles of 60°C/20s and 72°C/30 s
*FCRL4*	F R	GTGAGGGGTAACATCCACAAGC CTTCAGCCACGGAGCAGAC	148	95°C/30 s, 95°C/15.s, 40 cycles of 61°C/20s and 72°C/30 s
*GAPDH*	F R	GGACTCATGACCACAGTCCA CCAGTAGAGGCAGGGATGAT	199	95°C/30 s, 95°C/15.s, 40 cycles of 57.5°C/20s and 72°C/30 s

**Table 2:** Demographic and Laboratory Indexes of Kidney Transplant Recipients with and without Graft Rejection.

**Patient characteristics**	**Patients without rejection**	**Patients with rejection**
**Age (years), mean±SEM**	51.62 ± 10.60	50.95 ± 10.61
**Sex, n( %)**		
Female	7 (27)	1(15)
Male	19 (73)	5(85)
**Blood group, n( %)**		
A positive	8(30.8)	1(16.7)
B positive	5(19.2)	2(33.2)
AB positive A positive	1(15.4)	0(0)
O positive	8(30.8)	2(33.3)
O negative	1(3.8)	1(16.7)

**Table 3:** Genes Expression of Non-rejection, Rejected, and Control Groups at 1st, 3rd, and 7th Days of Kidney Post-transplantation

**Day**	**Groups**	**FCRL1**	**P-value**	**FCRL2**	**P-value**	**FCRL4**	**P-value**
**1st**	NR	1.313±0.39	0.0001	2.504±0.64	0.0001	0.683±0.2	0.0001
	R	1.421±1.09	0.0001	1.375±0.59	NS	1.615±0.54	NS
	C	2.198±0.68		3.25±0.55		2.83±0.66	
**3rd**	NR	1.841±0.50	0.0001	3.376±0.66	NS	1.815±0.50	0.0001
	R	1.157±0.72	0.04	3.064±2.2	NS	0.288±0.07	NS
	C	2.198±0.68		3.25±0.55		2.83±0.66	
**7th**	NR	1.59±0.44	0.0001	3.127±0.84	0.0001	1.00±0.31	0.0001
	R	1.04±0.35	NS	3.298±1.52	NS	0.34±0.1	NS
	C	2.198±0.68		3.25±0.55		2.83±0.66	

**NR: **Non-rejection; **R:** Rejection; **C:** Control; **NS:** not significant
